# Customized versus population birth weight charts for identification of newborns at risk of long-term adverse cardio-metabolic and respiratory outcomes: a population-based prospective cohort study

**DOI:** 10.1186/s12916-019-1424-4

**Published:** 2019-10-17

**Authors:** Jan S. Erkamp, Vincent W. V. Jaddoe, Annemarie G. M. G. J. Mulders, Eric A. P. Steegers, Irwin K. M. Reiss, Liesbeth Duijts, Romy Gaillard

**Affiliations:** 1 The Generation R Study Group, Erasmus MC, University Medical Center Rotterdam, P.O. Box 2040, 3000 CA Rotterdam, The Netherlands; 2 Department of Paediatrics, Erasmus MC, University Medical Center Rotterdam, Rotterdam, The Netherlands; 3 Department of Obstetrics & Gynaecology, Erasmus MC, University Medical Center Rotterdam, Rotterdam, The Netherlands; 4 Division of Neonatology, Department of Paediatrics, Erasmus MC, University Medical Center Rotterdam, Rotterdam, The Netherlands; 5 Division of Respiratory Medicine and Allergology, Department of Paediatrics, Erasmus MC, University Medical Center Rotterdam, Rotterdam, The Netherlands

**Keywords:** Birth weight, Charts, Child, Outcomes, Customization, Cardiovascular health, Respiratory health

## Abstract

**Background:**

Customized birth weight charts take into account physiological maternal characteristics that are known to influence fetal growth to differentiate between physiological and pathological abnormal size at birth. It is unknown whether customized birth weight charts better identify newborns at risk of long-term adverse outcomes than population birth weight charts. We aimed to examine whether birth weight classification according to customized charts is superior to population charts at identification of newborns at risk of adverse cardio-metabolic and respiratory health outcomes.

**Methods:**

In a population-based prospective cohort study among 6052 pregnant women and their children, we measured infant catch-up growth, overweight, high blood pressure, hyperlipidemia, liver steatosis, clustering of cardio-metabolic risk factors, and asthma at age 10. Small size and large size for gestational age at birth was defined as birth weight in the lowest or highest decile, respectively, of population or customized charts. Association with birth weight classification was assessed using logistic regression models.

**Results:**

Of the total of 605 newborns classified as small size for gestational age by population charts, 150 (24.8%) were reclassified as appropriate size for gestational age by customized charts, whereas of the total of 605 newborns classified as large size for gestational age by population charts, 129 (21.3%) cases were reclassified as appropriate size for gestational age by customized charts. Compared to newborns born appropriate size for gestational age, newborns born small size for gestational age according to customized charts had increased risks of infant catch-up growth (odds ratio (OR) 5.15 (95% confidence interval (CI) 4.22 to 6.29)), high blood pressure (OR 2.05 (95% CI 1.55 to 2.72)), and clustering of cardio-metabolic risk factors at 10 years (OR 1.66 (95% CI 1.18 to 2.34)). No associations were observed for overweight, hyperlipidemia, liver steatosis, or asthma. Newborns born large-size for gestational age according to customized charts had higher risk of catch-down-growth only (OR 3.84 (95% CI 3.22 to 4.59)). The direction and strength of the observed associations were largely similar when we used classification according to population charts.

**Conclusions:**

Small-size-for-gestational-age newborns seem to be at risk of long-term adverse cardio-metabolic health outcomes, irrespective of the use of customized or population birth weight charts.

## Background

Small size for gestational age (SGA) and large size for gestational age (LGA) are important risk factors for adverse perinatal outcomes and death [1]. Children born SGA or LGA also have increased risks of suboptimal growth, cardio-metabolic, and respiratory development throughout childhood, leading to increased risks of obesity, coronary heart disease, type 2 diabetes, and obstructive respiratory disease in later life [2, 3]. Usually, population birth weight charts, which take into account gestational age at birth and sex, are used to discriminate between SGA, appropriate size for gestational age (AGA), and LGA newborns [4]. Newborns classified as SGA or LGA by these population charts include those who have grown according to their physiological growth potential and end up constitutionally small or large at birth, and those who have fetal growth restriction or acceleration and end up pathologically small or large at birth. Maternal characteristics, such as age, height, body mass index (BMI), ethnicity and parity, and fetal sex are important determinants of fetal growth and cause non-pathological variation in birth weight [5, 6]. Customized charts take these physiological maternal and fetal characteristics into account for classification of normal and abnormal weight at birth [7, 8]. Customized charts may therefore be better able to distinguish constitutionally from pathologically small or large size for gestational age at birth newborns [9]. Previous studies assessing the superiority of customized over population charts to identify SGA and LGA newborns at risk of short-term adverse outcomes are scarce and show conflicting results [10–13].

We hypothesized that compared to population charts, customized charts can better identify newborns at risk of long-term adverse health outcomes. We examined in a population-based prospective cohort study among 6052 newborns the associations of SGA and LGA based on both customized and population charts for identification of newborns at risk of adverse growth patterns, cardio-metabolic, and respiratory risk factors in childhood.

## Methods

### Study design

This study was embedded in the Generation R Study, a population-based prospective cohort study from early pregnancy onwards in Rotterdam, the Netherlands [14]. The study has been approved by the local Medical Ethical Committee (MEC 198.782/2001/31). Written consent was obtained from all participating women. All pregnant women were enrolled between 2001 and 2005. Response rate at birth was 61%. Eight thousand eight hundred seventy-nine women enrolled during pregnancy. We excluded non-singleton live births (*n* = 246), participants without information on weight and gestational age at birth or maternal characteristics needed to generate customized charts (*n* = 2004), and children without long-term outcomes available (*n* = 577). The population for analysis comprised 6052 mothers and their children (Additional file 1, Figure S1). Additional file 2 contains a Strengthening the Reporting of Observational Studies in Epidemiology (STROBE) statement for the current study [15].

### Classification of birth weight by customized and population charts

Customized charts have been developed within our study cohort as described previously and include gestational age, fetal sex, maternal parity, age, height, weight, and ethnicity [7]. The pathological determinant maternal smoking was also used for the development of the customized charts because it has a substantial effect on fetal growth and birth weight and led to a more accurate regression model [7]. For the construction of a customized growth chart, the term for smoking was set to zero, whether the pregnant woman smoked or not. Hereby, non-smoking was used as reference category within our customized models. To calculate the customized birth weight percentile, we entered the maternal characteristics, fetal sex, and gestational age at birth for each newborn within our customized charts model and compared actual birth weight to the expected weight. For the population charts, we used gestational age adjusted weight charts modeled on the same population [7]. We calculated the birth weight percentile, by entering gestational age at birth for each newborn within our population charts’ model, and compared actual birth weight to the expected weight. The population chart only included gestational age and no other characteristics, which allows for the optimal comparison between the population charts and customized charts in which any difference in outcome would only be explained by the process of customization. The formulas for both the customized charts and population charts have been published previously [7]. If the observed birth weight for gestational age was < 10th or > 90th percentile of the customized or population chart, the newborn was classified as SGA or LGA respectively, otherwise AGA. We compared classifications according to customized and population charts and further defined customized and population only SGA and LGA newborns. “Customized only” SGA or LGA newborns are classified as AGA by population charts but reclassified as SGA or LGA by customized charts. “Population only” SGA or LGA newborns are classified as AGA by customized charts but reclassified as SGA or LGA by population charts. Mode of delivery, offspring sex, gestational age, weight, and APGAR score were obtained from medical records [16]. Preterm birth was defined as a gestational age of < 37 weeks at birth.

### Childhood growth, cardio-metabolic, and respiratory outcomes

Well-trained staff in the Community Health Centers obtained postnatal growth characteristics at the age of 12 months and was available for 4205 (69.5%) participants. Catch-up and catch-down growth for weight was defined as an increase or decrease of > 0.67 SD of weight from birth to 12 months of age [17]. This change represents the width of each percentile band on standard growth charts.

At the age of 10 years, children were invited for detailed measurements. We measured height and weight without shoes and heavy clothing. We calculated sex- and age-adjusted childhood BMI SDS based on Dutch reference growth charts (Growth Analyzer 4.0 Dutch Growth Research Foundation) and categorized BMI into normal, overweight, and obesity using the definition of Cole et al. [18, 19]. Total body fat and lean mass were measured with a dual-energy X-ray absorptiometry (DXA) scanner (iDXA, Ge-Lunar, 2008, Madison, WI, USA) using encore software version 13.6. Fat mass index (FMI) was calculated: fat mass (kg)/height(m)^2^. Children were scanned using a 3.0 Tesla MRI (Discovery MR750w, GE Healthcare, Milwaukee, WI, USA) using standard protocols [20]. Visceral fat volumes were generated by summing volumes and multiplying by the gravity of adipose tissue, 0.9 g/ml. Liver fat fraction was determined by the average mean signal intensities from four samples of at least 4 cm^2^ from the central portion of the hepatic volume. Liver steatosis was defined as liver fat fraction ≥ 5.0%. Blood pressure was measured four times in supine position, with 1-min intervals at the right brachial artery using the automatic sphygmomanometer Datascope Accutor Plus (Paramus, NK) [21]. The mean of the last three measurements was calculated to determine blood pressure. High blood pressure was defined as systolic or diastolic blood pressure > 90th percentile, using sex-, age-, and height-specific cut-points [22]. Non-fasting venous blood samples were collected to measure total cholesterol, high-density lipoprotein (HDL)-cholesterol, triglycerides, and insulin concentrations using Cobas 8000 analyzer (Roche, Almere, the Netherlands). Recommendations from National Cholesterol Education Program for children age 2–9 were used to define adverse levels of total cholesterol (> 5.1 mmol/l) [23]. For clustering of cardio-metabolic risk factors, we used the definition of childhood metabolic syndrome phenotype, which is having three or more of the following components: visceral fat mass > 75th percentile, systolic or diastolic blood pressure > 75th percentile, HDL-cholesterol < 25th percentile or triglycerides > 75th percentile, and insulin level > 75th percentile of our study population [24].

Forced expiratory volume in the first second (FEV_1_), forced vital capacity (FVC), FEV1:FVC, and forced expiratory flow after expiring 75% of FVC (FEF_75_) were measured by spirometry (MasterScreen-Pneumo, Jaeger Toennies (Viasys) CareFusion Netherlands) [25]. Measures were converted into sex-, height-, age-, and ethnicity-adjusted SDS according to the Global Lung Initiative reference data [26]. Asthma was defined as ever physician-diagnosed asthma at age 10, obtained by parental reported questionnaires.

### Statistical analyses

First, each newborn was classified into birth weight categories using both customized and population classifications. Descriptive data of birth weight categories were compared. Second, the percentages of newborns reclassified as SGA or LGA by customized charts only or population charts only were assessed and population characteristics were compared using one-way ANOVA for continuous and chi-square test for categorical variables. Third, we assessed the associations of SGA and LGA at birth according to both customized and population charts with adverse outcomes using linear and logistic regression models for continuous and categorical outcomes, respectively. Non-normally distributed variables were log-transformed, and SDS were calculated. For categorical outcomes, we calculated prevalences of adverse outcomes among SGA, AGA, and LGA newborns, by dividing the number of cases by the number of newborns in each birth weight category. Finally, we assessed the predictive performance of both classifications for the prediction of the risk of long-term adverse health outcomes among SGA and LGA newborns by calculating receiver operating characteristic (ROC) curves, the corresponding area under the curve, and sensitivity at a 90% specificity. We did not adjust our analyses for potentially confounding maternal characteristics, as customized classification already considers maternal characteristics and we were interested in comparing the classifications. All analyses were performed using the Statistical Package of Social Sciences version 24.0 for Windows (IBM Corp., Armonk, NY, USA).

## Results

### Population characteristics

Table 1 shows population characteristics. Compared to newborns classified as AGA by customized charts, newborns classified as SGA by customized charts more often had heavier mothers and their mothers more often smoked throughout pregnancy. They were more often born premature or with a low APGAR score. Newborns classified as LGA by customized charts more often had multiparous mothers, compared to newborns classified as AGA by customized charts. Compared to newborns classified as AGA by population charts, newborns classified as SGA by population charts more often had nulliparous mothers and mothers with a lower weight and their mothers more often smoked throughout pregnancy. They were also more often born premature or with a low APGAR score. Newborns classified as LGA by population charts had heavier mothers and mothers who were multiparous, compared to newborns classified as AGA by population charts.

**Table 1:** Maternal and birth characteristics of newborns classified as SGA, AGA, or LGA by customized and population birth weight classifications

	Customized classification^a^			Population classification^b^		
	Small size for gestational age	Appropriate size for gestational age	Large size for gestational age	Small size for gestational age	Appropriate size for gestational age	Large size for gestational age
	*n* = 605	*n* = 4842	*n* = 605	*n* = 605	*n* = 4842	*n* = 605
Maternal characteristics						
Age, median (95% range), years	30.5 (19.7 to 39.8)	30.6 (19.6 to 39.1)	30.7 (20.1 to 39.3)	29.7 (19.0 to 39.6)	30.5 (19.8 to 39.0)	31.6 (21.5 to 39.8)
Height, mean (SD) (cm)	166.2 (7.4)	167.7 (7.4)	169.3 (7.3)	164.3 (7.1)	167.7 (7.3)	170.6 (7.2)
Weight, mean (SD) (kg)	71.8 (16.3)	68.7 (12.3)	70.4 (12.9)	64.8 (12.5)	68.8 (12.4)	76.0 (14.6)
Body mass index, mean (SD) (kg/m^2^)	26 (5.6)	24.4 (4.1)	24.6 (4.4)	24.0 (4.4)	24.5 (4.2)	26.2 (5.1)
Obesity	123 (20.3)	485 (10.0)	60 (9.9)	58 (9.6)	491 (10.1)	119 (19.7)
Education, no. higher (%)	217 (37)	2137 (45)	306 (52)	206 (34.8)	2141 (45.2)	313 (52.8)
Ethnicity, no. (%) Dutch/European	332 (54.9)	2899 (59.9)	395 (65.3)	283 (46.8)	2911 (60.1)	432 (71.4)
Parity, no. Nulliparous (%)	348 (57.5)	2792 (57.7)	330 (54.5)	434 (71.7)	2787 (57.6)	249 (41.2)
Smoking, no. (%)						
None	440 (66.3)	3755 (73.9)	510 (79.6)	379 (65.2)	3466 (74.4)	462 (79.4)
Early pregnancy only	50 (7.5)	453 (8.9)	61 (9.5)	45 (7.7)	418 (9.0)	55 (9.5)
Continued	174 (26.2)	875 (17.2)	70 (10.9)	157 (27.0)	773 (16.6)	65 (11.2)
Birth characteristics						
Males, no. (%)	295 (42.8)	2665 (50.5)	390 (59.2)	242 (40.0)	2435 (50.3)	362 (59.8)
Gestational age, median (95% range) weeks	39.7 (32.0 to 42.3)	40.3 (36.3 to 42.3)	39.9 (36.2 to 42.1)	40.3 (36.3 to 42.4)	40.3 (36.3 to 42.4)	39.9 (36.0 to 42.0)
Birth weight, mean (SD) grams	2622 (483)	3440 (425)	4176 (396)	2581 (421)	3442 (416)	4230 (399)
Preterm birth, no. (%)	74 (12.2)	184 (3.8)	21 (3.5)	66 (10.9)	187 (3.9)	26 (4.3)
Cesarean delivery, no. (%)	107 (18.9)	467 (10.5)	94 (17.1)	102 (18.2)	478 (10.8)	88 (16.0)
Assisted delivery, no. (%)	75 (13.3)	644 (14.5)	75 (13.6)	59 (15.9)	651 (14.7)	54 (9.9)
APGAR score below 7 at 5 min, no. (%)	16 (2.8)	40 (0.9)	4 (0.9)	12 (2.1)	44 (0.9)	5 (0.9)

Values are median (95% range), mean (SD), and absolute numbers (%)

*SD* standard deviation

^a^SGA was defined as gestational age-adjusted birth weight < 10th percentile of the customized chart. AGA is defined as gestational age-adjusted birth weight > 10th and < 90th percentile according to the customized chart. LGA was defined as gestational age-adjusted birth weight > 90th percentile of the customized chart

^b^SGA was defined as gestational age-adjusted birth weight < 10th percentile of the population chart. AGA is defined as gestational age-adjusted birth weight > 10th and < 90th percentile of the population chart. LGA was defined as gestational age-adjusted birth weight > 90th percentile of the population chart

### Characteristics of newborns classified as SGA or LGA by customized or population charts only

Table 2 shows that of 605 newborns classified as SGA using population charts, 150 (24.8%) were reclassified as AGA using customized charts, whereas of 605 newborns classified as LGA using population charts, 129 (21.3%) cases were reclassified as AGA using customized charts. Mothers of newborns who were classified SGA as by customized charts only were likely to have higher BMIs and to be of Dutch or European ethnicity and were more often multiparous compared to mothers of newborns classified as SGA by both customized and population charts (Table 3). Newborns classified as SGA by customized charts only had a higher birth weight and were less likely to be born preterm and after assisted delivery compared to newborns classified as SGA by both customized and population charts. Mothers of newborns classified as LGA by customized charts only had lower age and BMI, were more often of Dutch or European ethnicity, and were more often nulliparous, compared to mothers of newborns classified as LGA by customized and population charts. Newborns classified as LGA by customized charts only had lower birth weight and were more likely born after assisted delivery compared to newborns classified as LGA by both charts. Mothers of newborns who were classified as SGA by population charts only were younger, less likely to be obese, of Dutch or European ethnicity, and to be nulliparous compared to mothers of newborns classified as SGA by both charts (Additional file 1: Table S1). Their newborns showed similar patterns to newborns classified as SGA by customized charts only. Mothers of newborns classified as LGA by population charts only were older, had higher BMI, and were more likely multiparous, and their newborns had lower birth weight compared to newborns classified as LGA using both charts.

**Table 2:** Agreement of classification of gestational age-adjusted birth weight by customized and population birth weight classifications

		Customized classification^a^		
		Small size for gestational age *n* = 605	Appropriate size for gestational age *n* = 4842	Large size for gestational age *n* = 605
Population classification^b^	Small size for gestational age *n* = 605	455 (75.2)	150 (24.8)	0 (0)
	Appropriate size for gestational age *n* = 4842	150 (24.8)	4563 (94.2)	129 (21.3)
	Large size for gestational age *n* = 605	0 (0)	129 (21.3)	476 (78.7)

Values are absolute cases (%)

^a^SGA was defined as gestational age-adjusted birth weight < 10th percentile of the customized chart. AGA is defined as gestational age-adjusted birth weight > 10th and < 90th percentile according to the customized chart. LGA was defined as gestational age-adjusted birth weight > 90th percentile of the customized chart

^b^SGA was defined as gestational age-adjusted birth weight < 10th percentile of the population chart. AGA is defined as gestational age-adjusted birth weight > 10th and < 90th percentile of the population chart. LGA was defined as gestational age-adjusted birth weight > 90th percentile of the population chart

**Table 3:** Maternal and birth characteristics of SGA or LGA newborns by customized charts only compared to newborns classified as SGA or LGA by both classifications

	Small size for gestational age			Large size for gestational age		
	Customized only^a^	Customized and population^b^	*p* value	Customized only^a^	Customized and population^b^	*p* value
	*n* = 150	*n* = 455		*n* = 129	*n* = 476	
Maternal characteristics						
Age, median (95% range), years	30.5 (20.4 to 39.0)	30.5 (19.6 to 40.3)	0.215	27.7 (19.9 to 36.0)	31.2 (20.7 to 40.0)	< 0.001
Height, mean (SD) (cm)	169.0 (7.8)	165.3 (7.0)	< 0.001	165.7 (6.4)	170.3 (7.2)	< 0.001
Weight, mean (SD) (kg)	85.4 (18.1)	67.3 (12.8)	< 0.001	60.8 (7.6)	72.9 (12.8)	< 0.001
Body mass index, mean (SD) (kg/m2)	30.0 (6.5)	24.6 (4.6)	< 0.001	22.2 (2.5)	25.2 (4.6)	< 0.001
Obesity, no. (%)	67 (44.7)	56 (12.3)	< 0.001	2 (1.6)	58 (12.2)	< 0.001
Education, no. higher (%)	51 (34.0)	166 (36.5)	0.582	57 (44.2)	249 (52.3)	0.102
Race/ethnicity, no. (%) Dutch or European	101 (67.3)	253 (55.6)	0.011	56 (43.4)	327 (68.7)	< 0.001
Parity, no. nulliparous (%)	50 (33.3)	298 (65.5)	< 0.001	108 (83.7)	222 (46.6)	< 0.001
Smoking, no. (%)			0.055			0.711
None	109 (75.2)	282 (64.5)		100 (80.0)	366 (79.2)	
Early pregnancy only	9 (6.2)	33 (7.6)		14 (11.2)	45 (9.7)	
Continued	27 (18.6)	122 (27.9)		11 (8.8)	51 (11.0)	
Birth characteristics						
Males, no. (%)	67 (44.7)	185 (40.7)	0.388	72 (55.8)	281 (59.0)	0.511
Gestational age, median (95% range), weeks	40.1 (32.6 to 42.4)	39.6 (31.9 to 42.3)	0.007	40.0 (36.4 to 42.4)	39.9 (36.1 to 42.0)	0.058
Birth weight, mean (SD) grams	2981 (412)	2503 (445)	< 0.001	3922 (319)	4245 (386)	< 0.001
Preterm birth, no. (%)	12 (8.0)	62 (13.6)	0.068	3 (2.3)	18 (3.8)	0.423
Cesarean delivery, no. (%)	19 (13.4)	88 (20.8)	0.051	20 (16.7)	74 (17.2)	0.897
Assisted delivery, no. (%)	9 (6.3)	66 (15.6)	0.005	27 (22.5)	48 (11.1)	0.001
APGAR score below 7 at 5 min, no. (%)	5 (3.4)	11 (2.5)	0.549	0 (0)	5 (1.1)	0.237

Values are median (95% range), mean (SD), and absolute numbers (%), and *p* values for comparison between population only and customized and population classification. Continuous variables were tested using ANOVA; categorical variables were tested using Chi^2^ tests

*SD* standard deviation

^a^As defined by the population birth weight classification, but appropriately sized according to customized birth weight classification

^b^As defined by the both customized and population birth weight classification

### Customized and population birth weight classification and childhood outcomes

**Fig. 1:**
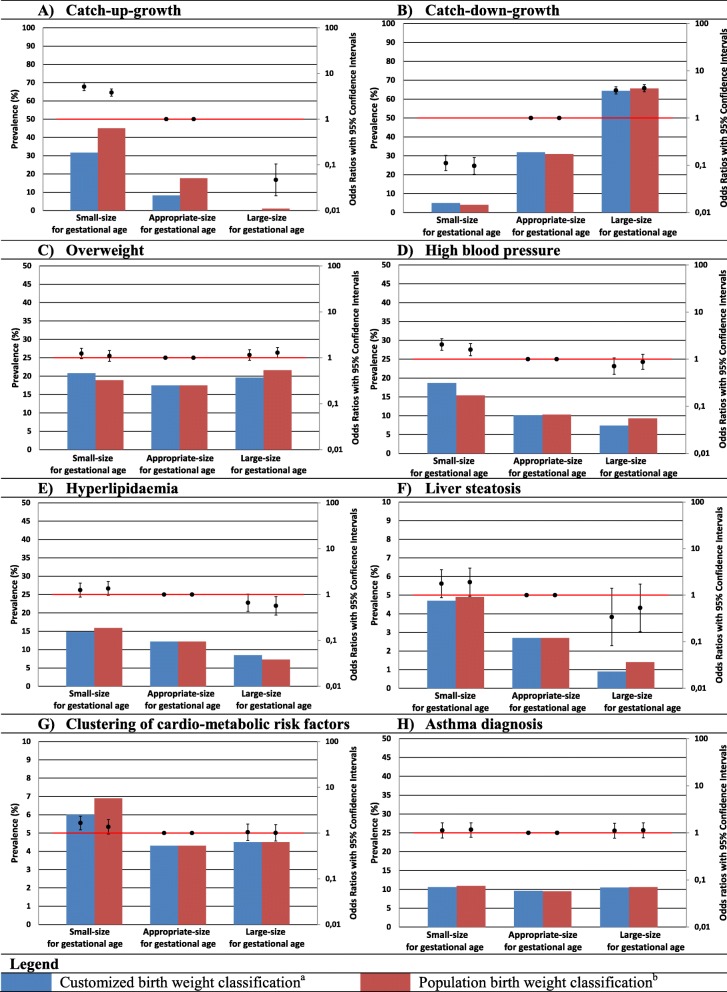
**a**–**h** Prevalence of birth weight classifications and their association with infant growth patterns and cardio-metabolic and respiratory outcomes at age 10. Bars are prevalence (%, left y-axis) and OR’s (95% CI, right y-axis). Reference groups for OR’s of customized and population classifications are newborns classified AGA according to the respective classification. Prevalences of adverse outcomes among SGA, AGA and LGA newborns were calculated by dividing the number of cases by the number of newborns in each birth weight category. Clustering of cardio-metabolic risk factors is defined as having three or more of the following components: visceral fat mass >75^th^ percentile; systolic or diastolic blood pressure >75^th^ percentile; HDL-cholesterol <25^th^ percentile or triglycerides >75^th^ percentile; and insulin level >75^th^ percentile of our study population. ^a^ SGA was defined as gestational age adjusted birth weight <10^th^ percentile of the customized chart. AGA is defined as gestational age adjusted birth weight >10^th^ and <90^th^ percentile of the customized chart. LGA was defined as gestational age adjusted birth weight >90^th^ percentile of the customized chart. ^b^ SGA was defined as gestational age adjusted birth weight <10^th^ percentile of the population birth weight chart. AGA is defined as gestational age adjusted birth weight >10^th^ and <90^th^ percentile the population chart. LGA was defined as gestational age adjusted birth weight >90^th^ percentile of the population chart

Based on customized charts, newborns classified as SGA had a higher risk of infant catch-up-growth compared to newborns classified as AGA (odds ratio (OR) 5.15 (95% confidence interval (CI) 4.22 to 6.29), Fig. 1a). Risk of catch-down growth was higher among newborns classified as LGA using customized charts, compared to newborns classified as AGA (OR 3.84 (95% CI 3.22 to 4.59), Fig. 1b). We observed similar associations when birth weight was classified using population charts.

**Fig. 2:**
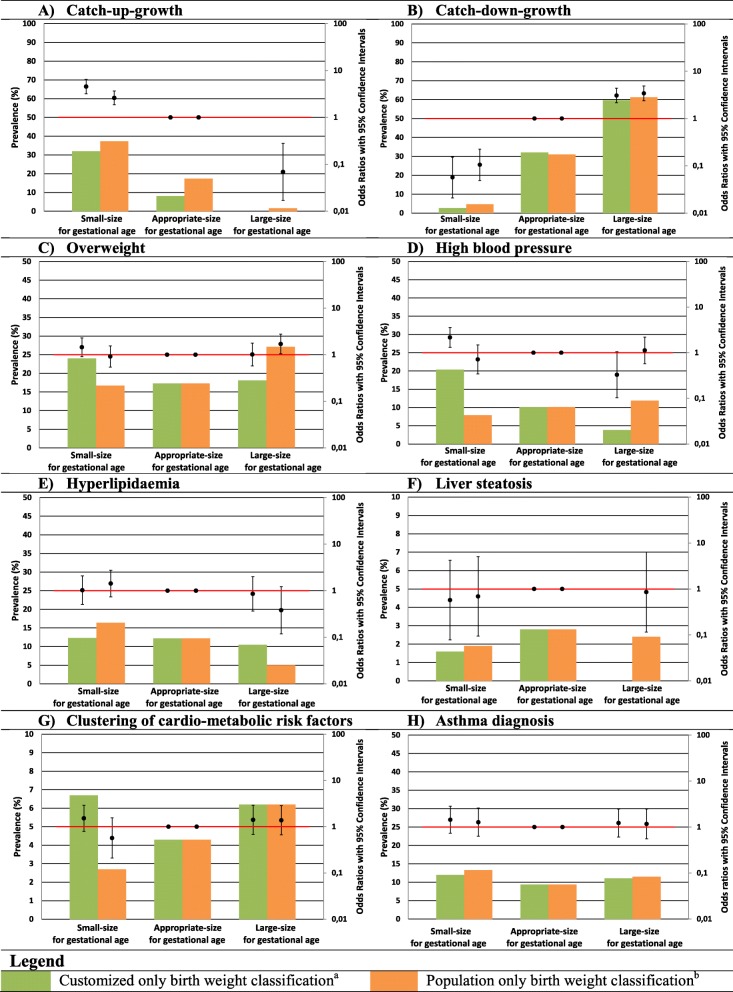
**a**–**h** Prevalence of customized only and population only birth weight classifications and their association with infant growth patterns and cardio-metabolic and respiratory outcomes at age 10. Bars are prevalence (%, left y-axis) and OR’s (95% CI, right y-axis). Reference groups for OR’s of customized and population classifications are newborns classified AGA according to both customized and population classification. Prevalences of adverse outcomes among SGA, AGA and LGA newborns were calculated by dividing the number of cases by the number of newborns in each birth weight category. Clustering of cardio-metabolic risk factors is defined as having three or more of the following components: visceral fat mass >75^th^ percentile; systolic or diastolic blood pressure >75^th^ percentile; HDL-cholesterol <25th percentile or triglycerides >75^th^ percentile; and insulin level >75^th^ percentile of our study population. ^a^ SGA was defined as gestational age adjusted birth weight <10^th^ percentile of the customized chart, but >10^th^ percentile according to the population chart. AGA is defined as gestational age adjusted birth weight >10^th^ and <90^th^ percentile of both the customized and population chart. LGA was defined as gestational age adjusted birth weight >90^th^ percentile of the customized chart, but not <90^th^ percentile according to the population chart. ^b^ SGA was defined as gestational age adjusted birth weight <10^th^ percentile of the population birth weight chart, but >10^th^ percentile according to the customized chart. AGA is defined as gestational age adjusted birth weight >10^th^ and <90^th^ percentile of both the population and customized chart. LGA was defined as gestational age adjusted birth weight >90th percentile of the population chart, but not <90^th^ percentile of the customized chart

Compared to newborns classified as AGA, newborns classified as SGA using customized charts had higher risks of high childhood blood pressure (OR 2.05 (95% CI 1.55 to 2.72)) and clustering of cardio-metabolic risk factors (OR 1.66 (95% CI 1.18 to 2.34)). They also tended to have higher risk of childhood overweight (OR 1.24 (95% CI 0.95 to 1.60)), hyperlipidemia (OR 1.25 (95% CI 0.88 to 1.79)), and liver steatosis (OR 1.77 (95% CI 0.88 to 3.54)), but these findings did not reach statistical significance (Fig. 1c–g). We observed similar associations when we used the population classification. Newborns classified as LGA using customized charts did not have increased risks of any adverse cardio-metabolic outcome. Newborns classified as LGA using population charts had higher risk of overweight (OR 1.29 (95% CI 1.00 to 1.67)) and a lower risk of hyperlipidemia (OR 0.57 (95% CI 0.36 to 0.90)) compared with newborns classified as AGA, but the differences in effect estimates compared to customized charts were very small. No associations of newborns classified as SGA or LGA using either classification with asthma was found. When we repeated the analyses among newborns classified as SGA or LGA by customized or population charts only, largely similar findings were observed. We only observed a slightly higher risk of high childhood blood pressure (OR 2.17 (95% CI 1.31 to 3.58)) among newborns classified as SGA by customized charts only compared to those classified as SGA by population charts only (Fig. 2). Additional file 1: Table S2 shows AUCs and derived sensitivities at a 90% specificity for both classifications for the risk of each long-term adverse health outcome. Both classifications had a poor to moderate ability to discriminate between those with and those without long-term adverse health outcomes with AUCs (95% CI) ranging from 0.51 (95% CI 0.48–0.54) and 0.51 (95% CI 0.48–0.54) for risk of childhood asthma diagnosis to 0.66 (95% CI 0.64–0.69) and 0.63 (95% CI 0.61–0.65) for risk of infant catch-up growth for customized and population charts, respectively.

Results presented in the supplementary materials show associations of birth weight using both customized and population classifications with continuously measured blood pressure, lipid, glucose and insulin concentrations, and lung function (Additional file 1: Table S3–S5). Altogether, no differences in associations were observed between effect estimates based on customized or population birth weight classifications.

## Discussion

Our findings suggest that newborns born SGA have increased risks of an adverse cardio-metabolic profile at school age. Newborns born LGA have an increased risk of catch-down growth. Similar associations were present for classifications using customized charts and population charts, which suggests that customized charts are not superior to population charts at identification of SGA newborns at increased risk of adverse cardio-metabolic and respiratory outcomes at later age.

### Interpretation of main findings

Birth weight is a strong determinant of neonatal health and health in later life [3]. Both experimental studies and large population studies have suggested that newborns born SGA or LGA as results of adverse fetal exposures experience developmental adaptations which put them at increased risks of adverse health outcomes in later life [3, 27]. Thus, identification of newborns with abnormal size at birth is important to identify individuals who might benefit from preventive strategies from early life onwards to prevent chronic diseases throughout the life course. Customized charts have been a topic of research for several decades as these charts may identify a higher proportion of newborns that are pathologically SGA or LGA and at increased risk of adverse outcomes, compared to population charts which may identify both constitutionally and pathologically SGA or LGA newborns [8, 9].

Previous studies mainly focused on the effects of customization on selecting newborns at risk for adverse perinatal outcomes. A meta-analysis including 20 studies comparing the effectiveness of customized versus population charts for prediction of adverse perinatal outcomes has shown similar effect estimates for associations of abnormal size at birth with intra-uterine fetal demise, neonatal intensive care unit admission, and neonatal and perinatal death [10]. A recent population-based linkage study among 979,912 singleton pregnancies in the UK between 1992 and 2010 assessed the predictive ability of non-customized versus partially customized birth weight centiles for the prediction of the risks of stillbirth, infant death, and neonatal morbidity. This study showed that partial customization of birth weight charts does not improve the prediction of these perinatal complications [28]. For the partial customization, maternal height, parity, and fetal sex were used. Contrary, analysis of data on live births and stillbirths in England and Wales between 2007 and 2012 from the Office of National Statistics suggested in areas that implemented customized charts, a decline in stillbirth rates of 19% occurred, while stillbirth rates remained the same in areas that did not implement customized charts [8, 29]. However, these findings need to be interpreted carefully, and causality cannot be established from these observational studies. Recently, a study across different countries in Europe, including the UK, performed between 2004 and 2010 showed that rates of stillbirths declined by an average of 17%. A large number of these countries did not implement the use of customized charts. Thus, in comparison by the overall decline in stillbirth rates in Europe, the difference in decline in stillbirth rates in areas with and without implementation of customized charts may be relatively small [29, 30]. To date, no studies compared the use of customized and population charts to identify newborns at risk of long-term adverse health outcomes. We observed that customized charts were not better at selecting newborns at risk of adverse long-term cardio-metabolic or respiratory outcomes compared to population charts. As the majority of SGA newborns are classified as such by both charts, the benefit of the customized classification would mainly be present among the small group of newborns reclassified as having a normal or abnormal size for gestational age at birth by the customized charts. Within our study, newborns classified as SGA by customized charts only did have higher risk of high blood pressure compared to AGA newborns and all SGA newborns, but these effects were not large enough to lead to a significant benefit of the use of customized over population charts. Thus, overall our study does not provide strong evidence for the use of customized charts to better identify newborns at risk of long-term adverse health outcomes. When we determined the accuracy of both classification methods for the prediction of individual risk of adverse outcomes, we observed a poor to moderate performance for both customized and population charts. This suggests that neither classification can be used for individual prediction of the risk for long-term adverse health outcomes based on classifying size at birth. However, the apparent increased risk of long-term adverse health outcomes among the group of SGA newborns, classified using either classification, suggests that on a population level this characteristic can be used for screening or prevention strategies, especially in combination with other prognostic factors.

There are several reasons why we might not observe strong differences in risks of long-term adverse outcomes between birth weights classified using customized or population charts. First, current customized birth weight charts have been criticized as they might not yet capture growth potential well enough to truly differentiate between pathologically and constitutionally SGA and LGA newborns [11, 31]. This would explain why we did not observe a clear benefit of customized charts over population charts for the identification of newborns at risk of adverse outcomes in later life. Future studies should determine whether customized charts can be improved by removal or addition of other parameters associated with fetal size and birth outcomes, such as parameters of placental vascular resistance or biomarkers [32, 33]. Second, it has been hypothesized that the observed stronger associations of abnormal fetal size or size at birth for gestational age based on customized charts with adverse perinatal outcomes could be explained by confounding, for example by preterm birth and maternal obesity [11]. In a previous study among 4095 women and their offspring, obesity and preterm birth were more prevalent among mothers of newborns classified as SGA using customized charts [11]. Associations with adverse outcomes attenuated after adjustment for maternal obesity and preterm birth. In our study, prevalence of obesity among mothers of newborns classified as SGA using customized charts was twice that of mothers of newborns classified as SGA using population charts. Among newborns classified as SGA using customized charts only, maternal obesity was even fourfold higher, which might explain a tendency for larger effect sizes for risk of childhood overweight, high blood pressure, and clustering of cardio-metabolic risk factors. Thus, small differences in effect estimates between associations of SGA classified by customized and population charts might be explained by confounding factors. Finally, we might not have found strong differences in risk of adverse outcomes between customized and population charts, because our population is relatively healthy. We did not have extreme cases of SGA or LGA, and the prevalence of long-term adverse health outcomes is low within our cohort. The potential advantage of the use of customized charts might be stronger among higher-risk populations. Further studies in these populations are needed.

Based on the findings in our study and the fact that population charts are easier to use and widely implemented, we would not recommend implementation of customized birth weight charts for identification of newborns at risk of long-term adverse health outcomes.

### Strengths and limitations

We had a prospective data collection from early pregnancy onwards and a large sample of 6052 newborns available with detailed childhood growth, cardio-metabolic, and respiratory measurements. Loss to follow-up could have reduced statistical power and led to biased effect estimates if associations differ between children included and not included in the analysis. We do not think this poses a problem within our study, as the aim of our study was to compare two classification methods. The non-response at baseline might have led to selection of a healthier population, which might affect the generalizability of our results to higher-risk populations. In clinical practice, often sex-specific population charts are used to classify abnormal size at birth weight. Given the aim of our study to specifically assess the effect of customization by major determinants of fetal growth, we constructed a population chart which included gestational age only to enable the most optimal comparison. By including fetal sex in the population chart, we could underestimate the effect of customized charts, as fetal sex is one of the major physiological determinants of fetal growth. If we had included fetal sex in our population charts, we expect similar or even weaker differences between the associations of abnormal size at birth with the risk of long-term adverse outcomes according to customized charts and population charts. Which maternal factors should be included in the customized charts also remains debatable. We included the pathological variable maternal smoking in the construction of the model to obtain a better fitted model. For the construction of a customized growth chart, the term for smoking was set to zero, whether the pregnant woman smoked or not, and thereby, non-smoking was used as reference category within our customized model for all women. This approach still allowed us to detect pathological fetal growth restriction due to maternal smoking during pregnancy. A similar approach may also be used for other pathological variables and further improve customized charts. Further studies are needed to explore whether customized charts which consider more maternal factors improve the classification of size at birth*.* Blood sample collection was performed in a non-fasting state at different time points in the day. Since glucose and insulin levels are sensitive towards carbohydrate intake and vary during the day, this may have led to non-differential misclassification and an underestimation of the observed effect estimates.

## Conclusion

SGA newborns seem to be at risk of long-term adverse cardio-metabolic health outcomes, irrespective of use of customized or population birth weight charts. Our results suggest that customized charts are not superior to population charts at selecting newborns at risk of adverse childhood growth, cardio-metabolic, and respiratory outcomes. Based on these findings, we do not recommend implementation of customized charts for selection of newborns at risk of long-term adverse outcomes.

## Supplementary information

**Additional file 1: Figure S1.** Population for analysis. **Table S1.** Maternal and birth characteristics of SGA and LGA newborns by population charts only compared to newborns classified SGA or LGA by both classifications. **Table S2.** Predictive power of customized and population charts for long-term adverse outcomes. **Table S3.** Body composition and blood pressure at 10 years among newborns classified SGA or LGA by customized and population charts. **Table S4.** Cholesterol, triglycerides, insulin and glucose at 10 years among newborns classified SGA or LGA by customized and population charts. **Table S5.** Measures of lung function at 10 years among newborns classified SGA or LGA by customized and population charts.

**Additional file 2.** STROBE statement.

## Data Availability

Data requests can be made to the secretariat of Generation R.

## Abbreviations

AGA: Appropriate size for gestational age
BMI: Body mass index
CI: Confidence interval
DXA: Dual-energy X-ray absorptiometry
FEF_75_: Forced expiratory flow after expiring 75% of forced vital capacity
FEV_1_: Forced expiratory volume in the first second
FMI: Fat mass index
FVC: Forced vital capacity
HDL: High-density lipoprotein
LGA: Large size for gestational age
MRI: Magnetic resonance imaging
OR: Odds ratio
SDS: Standard deviation score
SGA: Small size for gestational age

## References

[1] Pallotto EK, Kilbride HW (2006). Perinatal outcome and later implications of intrauterine growth restriction. Clin Obstet Gynecol.

[2] den Dekker HT, Jaddoe VWV, Reiss IK, de Jongste JC, Duijts L (2018). Fetal and infant growth patterns and risk of lower lung function and asthma. The generation R study. Am J Respir Crit Care Med.

[3] Gluckman PD, Hanson MA, Cooper C, Thornburg KL (2008). Effect of in utero and early-life conditions on adult health and disease. N Engl J Med.

[4] Press R (2008). Antenatal care: Routine care for the healthy pregnant woman. RCOG Press at the Royal College of Obsstetricians and Gynaecologists.

[5] Gaillard R, Rurangirwa AA, Williams MA, Hofman A, Mackenbach JP, Franco OH, Steegers EA, Jaddoe VW (2014). Maternal parity, fetal and childhood growth, and cardiometabolic risk factors. Hypertension.

[6] Gaillard R, Durmus B, Hofman A, Mackenbach JP, Steegers EA, Jaddoe VW (2013). Risk factors and outcomes of maternal obesity and excessive weight gain during pregnancy. Obesity (Silver Spring).

[7] Gaillard R, de Ridder MA, Verburg BO, Witteman JC, Mackenbach JP, Moll HA, Hofman A, Steegers EA, Jaddoe VW (2011). Individually customised fetal weight charts derived from ultrasound measurements: the Generation R Study. Eur J Epidemiol.

[8] Gardosi J, Francis A, Turner S, Williams M (2018). Customized growth charts: rationale, validation and clinical benefits. Am J Obstet Gynecol.

[9] Gardosi J, Chang A, Kalyan B, Sahota D, Symonds EM (1992). Customised antenatal growth charts. Lancet.

[10] Chiossi G, Pedroza C, Costantine MM, Truong VTT, Gargano G, Saade GR (2017). Customized vs population-based growth charts to identify neonates at risk of adverse outcome: systematic review and Bayesian meta-analysis of observational studies. Ultrasound Obstet Gynecol.

[11] Sovio U, Smith GCS (2018). The effect of customization and use of a fetal growth standard on the association between birthweight percentile and adverse perinatal outcome. Am J Obstet Gynecol.

[12] Larkin JC, Hill LM, Speer PD, Simhan HN (2012). Risk of morbid perinatal outcomes in small-for-gestational-age pregnancies: customized compared with conventional standards of fetal growth. Obstet Gynecol.

[13] Verkauskiene R, Figueras F, Deghmoun S, Chevenne D, Gardosi J, Levy-Marchal M (2008). Birth weight and long-term metabolic outcomes: does the definition of smallness matter?. Horm Res.

[14] Kooijman MN, Kruithof CJ, van Duijn CM, Duijts L, Franco OH, van IMH, de Jongste JC, Klaver CC, van der Lugt A, Mackenbach JP (2016). The Generation R Study: design and cohort update 2017. Eur J Epidemiol.

[15] von Elm E, Altman DG, Egger M, Pocock SJ, Gotzsche PC, Vandenbroucke JP, Initiative S (2008). The Strengthening the Reporting of Observational Studies in Epidemiology (STROBE) statement: guidelines for reporting observational studies. J Clin Epidemiol.

[16] Jaddoe VW, van Duijn CM, Franco OH, van der Heijden AJ, van Iizendoorn MH, de Jongste JC, van der Lugt A, Mackenbach JP, Moll HA, Raat H (2012). The Generation R Study: design and cohort update 2012. Eur J Epidemiol.

[17] Taal HR, Vd Heijden AJ, Steegers EA, Hofman A, Jaddoe VW (2013). Small and large size for gestational age at birth, infant growth, and childhood overweight. Obesity (Silver Spring).

[18] Fredriks AM, van Buuren S, Wit JM, Verloove-Vanhorick SP (2000). Body index measurements in 1996–7 compared with 1980. Arch Dis Child.

[19] Cole TJ, Bellizzi MC, Flegal KM, Dietz WH (2000). Establishing a standard definition for child overweight and obesity worldwide: international survey. BMJ.

[20] White T, Muetzel RL, El Marroun H, Blanken LME, Jansen P, Bolhuis K, Kocevska D, Mous SE, Mulder R, Jaddoe VWV (2018). Paediatric population neuroimaging and the Generation R Study: the second wave. Eur J Epidemiol.

[21] Wong SN, Tz Sung RY, Leung LC (2006). Validation of three oscillometric blood pressure devices against auscultatory mercury sphygmomanometer in children. Blood Press Monit.

[22] National High Blood Pressure Education Program Working Group on High Blood Pressure in C, Adolescents. The fourth report on the diagnosis, evaluation, and treatment of high blood pressure in children and adolescents. Pediatrics 2004;114(Supplement 2):555–576. 15286277

[23] National cholesterol Education Program (2006). Guidelines for lipid Management in Children and Adolescents.

[24] Gishti O, Gaillard R, Durmus B, Abrahamse M, van der Beek EM, Hofman A, Franco OH, de Jonge LL, Jaddoe VW (2015). BMI, total and abdominal fat distribution, and cardiovascular risk factors in school-age children. Pediatr Res.

[25] den Dekker HT, Sonnenschein-van der Voort AMM, de Jongste JC, Anessi-Maesano I, Arshad SH, Barros H, Beardsmore CS, Bisgaard H, Phar SC, Craig L (2016). Early growth characteristics and the risk of reduced lung function and asthma: a meta-analysis of 25,000 children. J Allergy Clin Immunol.

[26] Quanjer PH, Stanojevic S, Cole TJ, Baur X, Hall GL, Culver BH, Enright PL, Hankinson JL, Ip MS, Zheng J (2012). Multi-ethnic reference values for spirometry for the 3-95-yr age range: the global lung function 2012 equations. Eur Respir J.

[27] Barker DJ (2004). The developmental origins of adult disease. J Am Coll Nutr.

[28] Iliodromiti S, Mackay DF, Smith GC, Pell JP, Sattar N, Lawlor DA, Nelson SM (2017). Customised and noncustomised birth weight centiles and prediction of stillbirth and infant mortality and morbidity: a cohort study of 979,912 term singleton pregnancies in Scotland. PLoS Med.

[29] Gardosi J, Giddings S, Clifford S, Wood L, Francis A (2013). Association between reduced stillbirth rates in England and regional uptake of accreditation training in customised fetal growth assessment. BMJ Open.

[30] Zeitlin J, Mortensen L, Cuttini M, Lack N, Nijhuis J, Haidinger G, Blondel B, Hindori-Mohangoo AD, Euro-Peristat Scientific C (2016). Declines in stillbirth and neonatal mortality rates in Europe between 2004 and 2010: results from the Euro-Peristat project. J Epidemiol Community Health.

[31] Hutcheon JA, Zhang X, Platt RW, Cnattingius S, Kramer MS (2011). The case against customised birthweight standards. Paediatr Perinat Epidemiol.

[32] Broere-Brown ZA, Schalekamp-Timmermans S, Jaddoe VWV, Steegers EAP (2018). Fetal growth and placental growth factor umbilical cord blood levels. Fetal Diagn Ther.

[33] Conde-Agudelo A, Papageorghiou AT, Kennedy SH, Villar J (2013). Novel biomarkers for predicting intrauterine growth restriction: a systematic review and meta-analysis. BJOG.

