# Supplementary material for: Customized versus population birth weight charts for identification of newborns at risk of long-term adverse cardio-metabolic and respiratory outcomes: a population-based prospective cohort study
Source: BMC Med. 2019 Oct 17;17:186. doi: 10.1186/s12916-019-1424-4 (PMC6796410; doi:10.1186/s12916-019-1424-4)
Supplement: Supplementary file 1 — Additional file 1: Figure S1. Population for analysis. Table S1. Maternal and birth characteristics of SGA and LGA newborns by population charts only compared to newborns classified SGA or LGA by both classifications. Table S2. Predictive power of customized and population charts for long-term adverse outcomes. Table S3. Body composition and blood pressure at 10 years among newborns classified SGA or LGA by customized and population charts. Table S4. Cholesterol, triglycerides, insulin and glucose at 10 years among newborns classified SGA or LGA by customized and population charts. Table S5. Measures of lung function at 10 years among newborns classified SGA or LGA by customized and population charts. [file 12916_2019_1424_MOESM1_ESM.docx]

**SUPPLEMENTAL MATERIAL**

| **Page** |  |
| --- | --- |
| 2 | **Figure S1.** Population for analysis |
| 3 | **Table S1.** Maternal and birth characteristics of SGA and LGA newborns by population charts only compared to newborns classified SGA or LGA by both classifications. |
| 4 | **Table S2.** Predictive power of customized and population charts for long-term adverse outcomes |
| 5 | **Table S3.** Body composition and blood pressure at 10 years among newborns classified SGA or LGA by customized and population charts |
| 6 | **Table S4.** Cholesterol, triglycerides, insulin and glucose at 10 years among newborns classified SGA or LGA by customized and population charts |
| 7 | **Table S5.** Measures of lung function at 10 years among newborns classified SGA or LGA by customized and population charts |

**Figure S1.** Flowchart population for analysis

**n = 8879**Participants enrolled during pregnancy.

n = 246 excluded due to non singleton live births

**n = 8633**Singleton live births.

n = 2004 excluded due to no gestational age, birth weight or customized classification available.

**n** **= 6629**Participants with singleton live births with information available for customized birth weight classification eligible for present study

n = 577 excluded due to no information on any long-term outcome measure.

**Total population for analysis = 6052**

Participants with singleton live births with information available for customized birth weight classification eligible for present study and long-term outcomes

**Growth outcomes**
0-12 months n = 4205
10 years n = 4063

**Cardio-metabolic outcomes at age 10**
General fat mass n = 4013
Visceral and liver fat n = 2261
Blood pressure n = 3922
Cholesterol, triglycerides, insulin and glucose n = 3227  **Respiratory outcomes at age 10**
Lung function n = 3785
Asthma diagnosis n = 3499

**Table S1.** Maternal and birth characteristics of SGA and LGA newborns by population charts only compared to newborns classified SGA or LGA by both classifications

|  | **Small-size for gestational age** | |  | **Large-size for gestational age** | |  |
| --- | --- | --- | --- | --- | --- | --- |
|  | Population only^a^ | Customized and population^b^ | p-value | Population only^a^ | Customized and population^b^ | p-value |
|  | n=150 | n=455 |  | n=129 | n=476 |  |
| **Maternal characteristics** |  |  |  |  |  |  |
| Age, median (95% range), years | 26.8(17.3 to 38.6) | 30.5(19.6 to 40.3) | <0.001 | 32.9(21.7 to 39.6) | 31.2(20.7 to 40.0) | 0.002 |
| Height, mean (SD) (cm) | 161.4(6.4) | 165.3(7.0) | <0.001 | 171.6(7.3) | 170.3(7.2) | 0.064 |
| Weight, mean (SD) (kg) | 57.2(7.4) | 67.3(12.9) | <0.001 | 87.5(15.2) | 72.9(12.8) | <0.001 |
| Body Mass Index, mean (SD) (kg/m2) | 22.0(2.6) | 24.6(4.6) | <0.001 | 29.8(5.5) | 25.2(4.6) | <0.001 |
| Obesity, No. (%) | 2(1.3) | 56(12.3) | <0.001 | 61(47.3) | 58(12.2) | <0.001 |
| Education, No Higher (%) | 40(26.7) | 166(36.5) | 0.028 | 64(49.6) | 249(52.3) | 0.586 |
| Race / Ethnicity, No. (%) Dutch or European | 46(30.7) | 253(55.6) | <0.001 | 99(76.7) | 327(68.7) | 0.076 |
| Parity, No. nulliparous (%) | 136(90.7) | 298(65.5) | <0.001 | 27(20.9) | 222(46.6) | <0.001 |
| Smoking, No. (%) |  |  | 0.690 |  |  | 0.887 |
| -None | 97(67.4) | 282(64.5) |  | 96(80.0) | 366(79.2) |  |
| -Early-pregnancy only | 12(8.3) | 33(7.6) |  | 10(8.3) | 45(9.7) |  |
| -Continued | 35(24.3) | 122(27.9) |  | 14(11.7) | 51(11.0) |  |
|  |  |  |  |  |  |  |
| **Birth characteristics** |  |  |  |  |  |  |
| Males, No. (%) | 57(38.0) | 185(40.7) | 0.564 | 81(62.8) | 281(59.0) | 0.440 |
| Gestational age, median (95% range), weeks | 40.1(36.5 to 42.6) | 39.6(31.9 to 42.3) | <0.001 | 40.1(34.1 to 42.0) | 39.9(36.1 to 42.0) | 0.940 |
| Birth weight, mean (SD) grams | 2816(201) | 2504(445) | <0.001 | 4060(411) | 4245(386) | <0.001 |
| Preterm birth, No. (%) | 4(2.7) | 62(13.6) | <0.001 | 8(6.2) | 18(3.8) | 0.229 |
| Caesarean delivery, No. (%) | 14(10.3) | 88(20.8) | 0.006 | 14(11.9) | 74(17.2) | 0.164 |
| Assisted delivery, No. (%) | 23(16.9) | 66(15.6) | 0.717 | 6(5.1) | 48(11.1) | 0.050 |
| Apgar score below 7 at 5 minutes, No. (%) | 1(0.7) | 11(2.5) | 0.175 | 0 | 5(1.1) | 0.243 |

Abbreviations: SD: Standard deviation; Values are median (95% range), mean (SD) and absolute numbers (%), and p-values for comparison between population only and customized and population classification. Continuous variables were tested using ANOVA, categorical variables were tested using Chi^2^ tests.

^a^ As defined by the population birth weight classification, but appropriately sized according to customized birth weight classification.
^b^ As defined by the both customized and population birth weight classification.

**Table S2.** Predictive power of customized and population charts for long-term adverse outcomes

|  |  | **Customized charts** | **Population charts** |
| --- | --- | --- | --- |
|  |  |  |  |
| Catch-up growth | AUC (95% CI) | 0.66 (0.64 to 0.69) | 0.63 (0.61 to 0.65) |
|  | Sensitivity | 32.4 | 24.1 |
| Catch-down growth | AUC (95% CI) | 0.62 (0.61 to 0.64) | 0.63 (0.61 to 0.64) |
|  | Sensitivity | 19.8 | 20.7 |
| Overweight | AUC (95% CI) | 0.52 (0.49 to 0.54) | 0.52 (0.49 to 0.54) |
|  | Sensitivity | 11.2 | 11.6 |
| High blood pressure | AUC (95% CI) | 0.55 (0.52 to 0.58) | 0.53 (0.50 to 0.56) |
|  | Sensitivity | 16.9 | 14.1 |
| Hyperlipidemia | AUC (95% CI) | 0.53 (0.49 to 0.56) | 0.54 (0.50 to 0.57) |
|  | Sensitivity | 12.0 | 12.6 |
| Liver steatosis | AUC (95% CI) | 0.56 (0.49 to 0.63) | 0.55 (0.48 to 0.63) |
|  | Sensitivity | 16.1 | 16.1 |
| Clustering of cardio-metabolic riskfactors^a^ | AUC (95% CI) | 0.53 (0.49 to 0.56) | 0.54 (0.50 to 0.57) |
|  | Sensitivity | 15.2 | 13.0 |
| Asthma diagnosis | AUC (95% CI) | 0.51 (0.48 to 0.54) | 0.51 (0.48 to 0.54) |
|  | Sensitivity | 10.3 | 10.6 |

Values are area under the receiver operating characteristic curve (95% Confidence Intervals), sensitivity(%) at 90% specificity.

^a^Clustering of cardio-metabolic risk factors is defined as having three or more of the following components: visceral fat mass >75^th^ percentile; systolic or diastolic blood pressure >75^th^ percentile; HDL-cholesterol <25^th^ percentile or triglycerides >75^th^ percentile; and insulin level >75^th^ percentile of our study population**.**

**Table S3.** Body composition and blood pressure at 10 years among newborns classified SGA or LGA by customized and population charts

|  | **Fat mass index** | n= | **Systolic blood pressure** | n= | **Diastolic blood pressure** | n= |
| --- | --- | --- | --- | --- | --- | --- |
| **Customized**^a^ |  |  |  |  |  |  |
| Small-size for gestational age | 0.23 (0.12 to 0.33) | 387 | 0.21 (0.10 to 0.31) | 379 | 0.15 (0.04 to 0.26) | 379 |
| Appropriate-size for gestational age | *ref* | 3238 | *ref* | 3164 | *ref* | 3164 |
| Large-size for gestational age | -0.07 (-0.18 to 0.04) | 388 | -0.07 (-0.17 to 0.04) | 379 | -0.08 (-0.19 to 0.03) | 379 |
| **Population^b^** |  |  |  |  |  |  |
| Small-size for gestational age | 0.22 (0.12 to 0.33) | 388 | 0.05 (-0.05 to 0.16) | 382 | 0.05 (-0.06 to 0.16) | 382 |
| Appropriate-size for gestational age | *ref* | 3236 | *ref* | 3157 | *ref* | 3157 |
| Large-size for gestational age | -0.03 (-0.13 to 0.08) | 389 | -0.01 (-0.12 to 0.09) | 383 | -0.03 (-0.14 to 0.08) | 383 |
| **­­­Customized only^c^** |  |  |  |  |  |  |
| Small-size for gestational age | 0.22(0.02 to 0.42) | 100 | 0.38(0.18 to 0.58) | 98 | 0.34(0.14 to 0.54) | 98 |
| Appropriate-size for gestational age | *ref* | 3053 | *ref* | 2979 | *ref* | 2979 |
| Large-size for gestational age | -0.05(-0.27 to 0.17) | 83 | -0.18 (-0.40 to 0.04) | 80 | -0.10(-0.32 to 0.13) | 80 |
| **Population only^d^** |  |  |  |  |  |  |
| Small-size for gestational age | 0.19(-0.01 to 0.39) | 101 | -0.18(-0.38 to 0.02) | 101 | -0.03(-0.22 to 0.17) | 101 |
| Appropriate-size for gestational age | *ref* | 3053 | *ref* | 2979 | *ref* | 2979 |
| Large-size for gestational age | 0.14(-0.08 to 0.35) | 84 | 0.10(-0.12 to 0.31) | 84 | 0.16(-0.06 to 0.37) | 84 |

Values are SDS (95% CI). N = number of measurements available.

^a^ SGA was defined as gestational age adjusted birth weight <10^th^ percentile of the customized chart. AGA is defined as gestational age adjusted birth weight >10^th^ and <90^th^ percentile according to the customized chart. LGA was defined as gestational age adjusted birth weight >90^th^ percentile of the customized chart.
^b^ SGA was defined as gestational age adjusted birth weight <10^th^ percentile of the population chart. AGA is defined as gestational age adjusted birth weight >10^th^ and <90^th^ percentile of the population chart. LGA was defined as gestational age adjusted birth weight >90^th^ percentile of the population chart.
^c^ SGA was defined as gestational age adjusted birth weight <10^th^ percentile of the customized chart, but >10^th^ percentile according to the population chart. AGA is defined as gestational age adjusted birth weight >10^th^ and <90^th^ percentile of both the customized and population chart. LGA was defined as gestational age adjusted birth weight >90^th^ percentile of the customized chart, but not <90^th^ percentile according to the population chart.
^d^ SGA was defined as gestational age adjusted birth weight <10^th^ percentile of the population birth weight chart, but >10^th^ percentile according to the customized chart. AGA is defined as gestational age adjusted birth weight >10^th^ and <90^th^ percentile of both the population and customized chart. LGA was defined as gestational age adjusted birth weight >90^th^ percentile of the population chart, but not <90^th^ percentile of the customized chart.

**Table S4.** Cholesterol, triglycerides, insulin and glucose at 10 years among newborns classified SGA or LGA by customized and population charts

|  | **Total cholesterol** | n= | **HDL cholesterol** | n= | **Triglycerides** | n= | **Insulin** | n= | **Glucose** | n= |
| --- | --- | --- | --- | --- | --- | --- | --- | --- | --- | --- |
| **Customized**^a^ |  |  |  |  |  |  |  |  |  |  |
| SGA | 0.04 (-0.08 to 0.17) | 277 | -0.07 (-0.20 to 0.05) | 276 | 0.17 (0.04 to 0.29) | 274 | 0.15 (0.03 to 0.27) | 277 | -0.06 (-0.19 to 0.06) | 277 |
| AGA | *ref* | 2274 | *ref* | 2275 | *ref* | 2267 | *ref* | 2270 | *ref* | 2275 |
| LGA | -0.13 (-0.26 to -0.01) | 284 | 0.03 (-0.09 to 0.15) | 284 | -0.04 (-0.17 to 0.08) | 284 | -0.05 (-0.17 to 0.08) | 283 | 0.05 (-0.18 to 0.07) | 283 |
| **Population^b^** |  |  |  |  |  |  |  |  |  |  |
| SGA | 0.10 (-0.03 to 0.23) | 271 | -0.05 (-0.17 to 0.08) | 271 | 0.22 (0.10 to 0.35) | 267 | 0.17 (0.04 to 0.29) | 271 | -0.08 (-0.21 to 0.05) | 271 |
| AGA | *ref* | 2277 | *ref* | 2277 | *ref* | 2271 | ref | 2273 | *ref* | 2278 |
| LGA | -0.20 (-0.32 to 0.08) | 287 | 0.00 (-0.11 to 0.14) | 287 | -0.03 (-0.15 to 0.09) | 287 | -0.01 (-0.14 to 0.11) | 286 | -0.01 (-0.14 to 0.11) | 286 |
| **Customized only^c^** |  |  |  |  |  |  |  |  |  |  |
| SGA | -0.09(-0.32 to 0.15) | 73 | -0.03(-0.26 to 0.21) | 72 | 0.04(-0.20 to 0.27) | 72 | -0.03(-0.26 to 0.20) | 73 | -0.12(-0.35 to 0.11) | 73 |
| AGA | *ref* | 2147 | *ref* | 2148 | *ref* | 2142 | ref | 2143 | *ref* | 2148 |
| LGA | 0.06(-0.20 to 0.33) | 57 | 0.14(-0.26 to 0.21) | 57 | -0.04(-0.30 to 0.22) | 57 | -0.11(-0.37 to 0.15) | 57 | -0.23(-0.49 to 0.03) | 57 |
| **Population only^d^** |  |  |  |  |  |  |  |  |  |  |
| SGA | 0.13(-0.11 to 0.38) | 67 | 0.08(-0.16 to 0.32) | 67 | 0.24(-0.00 to 0.49) | 65 | 0.02(-0.23 to 0.26) | 67 | -0.21(-0.46 to 0.03) | 67 |
| AGA | *ref* | 2147 | *ref* | 2148 | *ref* | 2142 | ref | 2143 | *ref* | 2148 |
| LGA | -0.25(-0.51 to 0.01) | 60 | 0.01(-0.25 to 0.27) | 60 | 0.01(-0.25 to 0.26) | 60 | 0.03(-0.23 to 0.29) | 60 | *-0.05(-0.30 to 0.21)* | 60 |

Abbreviations: SGA: Small-size for gestational age; AGA: Appropriate-size for gestational age; LGA: Large-size for gestational age.
Values are SDS (95% CI). N = number of measurements available.

^a^ SGA was defined as gestational age adjusted birth weight <10^th^ percentile of the customized chart. AGA is defined as gestational age adjusted birth weight >10^th^ and <90^th^ percentile according to the customized chart. LGA was defined as gestational age adjusted birth weight >90^th^ percentile of the customized chart.
^b^ SGA was defined as gestational age adjusted birth weight <10^th^ percentile of the population chart. AGA is defined as gestational age adjusted birth weight >10^th^ and <90^th^ percentile of the population chart. LGA was defined as gestational age adjusted birth weight >90^th^ percentile of the population chart.
^c^ SGA was defined as gestational age adjusted birth weight <10^th^ percentile of the customized chart, but >10^th^ percentile according to the population chart. AGA is defined as gestational age adjusted birth weight >10^th^ and <90^th^ percentile of both the customized and population chart. LGA was defined as gestational age adjusted birth weight >90^th^ percentile of the customized chart, but not <90^th^ percentile according to the population chart.
^d^ SGA was defined as gestational age adjusted birth weight <10^th^ percentile of the population birth weight chart, but >10^th^ percentile according to the customized chart. AGA is defined as gestational age adjusted birth weight >10^th^ and <90^th^ percentile of both the population and customized chart. LGA was defined as gestational age adjusted birth weight >90^th^ percentile of the population chart, but not <90^th^ percentile of the customized chart.

**Table S5.** Measures of lung function at 10 years among newborns classified SGA or LGA by customized and population charts

|  | **FEV1** | n= | **FEV1:FVC** | n= | **FEF75** | n= |
| --- | --- | --- | --- | --- | --- | --- |
| **Customized**^a^ |  |  |  |  |  |  |
| Small-size for gestational age | -0.19 (-0.30 to -0.09) | 364 | 0.02 (-0.09 to 0.13) | 364 | -0.02 (-0.12 to 0.08) | 365 |
| Appropriate-size for gestational age | *ref* | 3048 | *ref* | 3048 | *ref* | 3051 |
| Large-size for gestational age | 0.13 (0.02 to 0.24) | 368 | -0.02 (-0.12 to 0.09) | 368 | 0.04 (-0.06 to 0.14) | 368 |
| **Population^b^** |  |  |  |  |  |  |
| Small-size for gestational age | -0.18 (-0.29 to -0.07) | 364 | -0.06 (-0.17 to 0.05) | 364 | -0.06 (-0.16 to 0.04) | 364 |
| Appropriate-size for gestational age | *ref* | 3052 | *ref* | 3052 | *ref* | 3055 |
| Large-size for gestational age | 0.07 (-0.03 to 0.18) | 364 | -0.04 (-0.14 to 0.07) | 364 | 0.01 (-0.11 to 0.09) | 365 |
| **Customized only^c^** |  |  |  |  |  |  |
| Small-size for gestational age | -0.20(-0.41 to 0.00) | 93 | 0.15(-0.05 to 0.35) | 93 | 0.11(-0.08 to 0.30) | 94 |
| Appropriate-size for gestational age | *ref* | 2880 | *ref* | 2880 | *ref* | 2882 |
| Large-size for gestational age | 0.31(0.09 to 0.53) | 79 | 0.16(-0.05 to 0.38) | 79 | 0.24(0.03 to 0.45) | 79 |
| **Population only^d^** |  |  |  |  |  |  |
| Small-size for gestational age | -0.13(-0.34 to 0.07) | 93 | -0.13(-0.33 to 0.07) | 93 | -0.01(-0.21 to 0.18) | 93 |
| Appropriate-size for gestational age | *ref* | 2880 | *ref* | 2880 | *ref* | 2880 |
| Large-size for gestational age | 0.06(-0.17 to 0.29) | 75 | 0.12(-0.11 to 0.34) | 75 | 0.04(-0.17 to 0.25) | 76 |

Abbreviations: FEV1: Forced expiratory volume in the first second; FVC: Forced vital capacity; FEF75: Forced expiratory flow at 75% of FVC.
Values are SDS (95% CI). N = number of measurements available.
 ^a^ SGA was defined as gestational age adjusted birth weight <10^th^ percentile of the customized chart. AGA is defined as gestational age adjusted birth weight >10^th^ and <90^th^ percentile according to the customized chart. LGA was defined as gestational age adjusted birth weight >90^th^ percentile of the customized chart.
^b^ SGA was defined as gestational age adjusted birth weight <10^th^ percentile of the population chart. AGA is defined as gestational age adjusted birth weight >10^th^ and <90^th^ percentile of the population chart. LGA was defined as gestational age adjusted birth weight >90^th^ percentile of the population chart.
^c^ SGA was defined as gestational age adjusted birth weight <10^th^ percentile of the customized chart, but >10^th^ percentile according to the population chart. AGA is defined as gestational age adjusted birth weight >10^th^ and <90^th^ percentile of both the customized and population chart. LGA was defined as gestational age adjusted birth weight >90^th^ percentile of the customized chart, but not <90^th^ percentile according to the population chart.
^d^ SGA was defined as gestational age adjusted birth weight <10^th^ percentile of the population birth weight chart, but >10^th^ percentile according to the customized chart. AGA is defined as gestational age adjusted birth weight >10^th^ and <90^th^ percentile of both the population and customized chart. LGA was defined as gestational age adjusted birth weight >90^th^ percentile of the population chart, but not <90^th^ percentile of the customized chart.
