# Supplementary material for: Customized versus population birth weight charts for identification of newborns at risk of long-term adverse cardio-metabolic and respiratory outcomes: a population-based prospective cohort study
Source: BMC Med. 2019 Oct 17;17:186. doi: 10.1186/s12916-019-1424-4 (PMC6796410; doi:10.1186/s12916-019-1424-4)
Supplement: Supplementary file 2 — Additional file 2. STROBE statement. [file 12916_2019_1424_MOESM2_ESM.doc]

STROBE Statement—Checklist of items that should be included in reports of ***cohort studies***

|  | Item No | Recommendation |
| --- | --- | --- |
| **Title and abstract** | 1 | (*a*) Indicate the study’s design with a commonly used term in the title or the abstract  *Abstract (page 2, line 34)* |
|  |  | (*b*) Provide in the abstract an informative and balanced summary of what was done and what was found  *Abstract (page 2, lines 28-53)* |
| Introduction | | |
| Background/rationale | 2 | Explain the scientific background and rationale for the investigation being reported  *Introduction (page 3, lines 60-76)* |
| Objectives | 3 | State specific objectives, including any prespecified hypotheses  *Introduction (page 3, lines 77-81)* |
| Methods | | |
| Study design | 4 | Present key elements of study design early in the paper  *Study design (page 5, lines 86-95)* |
| Setting | 5 | Describe the setting, locations, and relevant dates, including periods of recruitment, exposure, follow-up, and data collection  *Methods (page 5, lines 86-177)* |
| Participants | 6 | (*a*) Give the eligibility criteria, and the sources and methods of selection of participants. Describe methods of follow-up  *Methods (page 5, lines 86-177)* |
|  |  | (*b*) For matched studies, give matching criteria and number of exposed and unexposed  *Not applicable.* |
| Variables | 7 | Clearly define all outcomes, exposures, predictors, potential confounders, and effect modifiers. Give diagnostic criteria, if applicable.  *(page 5-7, lines 98 -158)* |
| Data sources/ measurement | 8* | For each variable of interest, give sources of data and details of methods of assessment (measurement). Describe comparability of assessment methods if there is more than one group  *(page 5-7, lines 98 -158)* |
| Bias | 9 | Describe any efforts to address potential sources of bias |
| Study size | 10 | Explain how the study size was arrived at  *Study design (page 5, lines 86-95)* |
| Quantitative variables | 11 | Explain how quantitative variables were handled in the analyses. If applicable, describe which groupings were chosen and why  *Statistical analyses (page 5-7, lines 98 -158)* |
| Statistical methods | 12 | (*a*) Describe all statistical methods, including those used to control for confounding *Statistical analyses* (*page 7-8, lines 161-177)* |
|  |  | (*b*) Describe any methods used to examine subgroups and interactions  *Statistical analyses* (*page 7-8, lines 161-177)* |
|  |  | (*c*) Explain how missing data were addressed (p*age 7-8, lines 161-177)* |
|  |  | (*d*) If applicable, explain how loss to follow-up was addressed  (*page 7-8, lines 161-177)* |
|  |  | (*e*) Describe any sensitivity analyses (*page 7-8, lines 161-177)* |
| Results | | |
| Participants | 13* | (a) Report numbers of individuals at each stage of study—eg numbers potentially eligible, examined for eligibility, confirmed eligible, included in the study, completing follow-up, and analysed  *Figure S1* |
|  |  | (b) Give reasons for non-participation at each stage *Figure S1* |
|  |  | (c) Consider use of a flow diagram *Figure S1* |
| Descriptive data | 14* | (a) Give characteristics of study participants (eg demographic, clinical, social) and information on exposures and potential confounders *Table 1 (page 9)* |
|  |  | (b) Indicate number of participants with missing data for each variable of interest *Figure S1* |
|  |  | (c) Summarise follow-up time (eg, average and total amount) *Study design (page 5, lines 86-95)* |
| Outcome data | 15* | Report numbers of outcome events or summary measures over time |
| Main results | 16 | (*a*) Give unadjusted estimates and, if applicable, confounder-adjusted estimates and their precision (eg, 95% confidence interval). Make clear which confounders were adjusted for and why they were included *Figure 1, Figure 2* |
|  |  | (*b*) Report category boundaries when continuous variables were categorized |
|  |  | (*c*) If relevant, consider translating estimates of relative risk into absolute risk for a meaningful time period |
| Other analyses | 17 | Report other analyses done—eg analyses of subgroups and interactions, and sensitivity analyses (*page 7-8, lines 161-177)* |
| Discussion | | |
| Key results | 18 | Summarise key results with reference to study objectives  *Discussion (page 14, lines 262-266)* |
| Limitations | 19 | Discuss limitations of the study, taking into account sources of potential bias or imprecision. Discuss both direction and magnitude of any potential bias  *Strengths and limitations (page 17, lines 345 -371)* |
| Interpretation | 20 | Give a cautious overall interpretation of results considering objectives, limitations, multiplicity of analyses, results from similar studies, and other relevant evidence  *Discussion (page 14, lines 268 – 343)* |
| Generalisability | 21 | Discuss the generalisability (external validity) of the study results  *Strengths and limitations (page 17, lines 345 -371)* |
| Other information | | |
| Funding | 22 | Give the source of funding and the role of the funders for the present study and, if applicable, for the original study on which the present article is based  *Funding (page 19, lines 409 – 417)* |

*Give information separately for exposed and unexposed groups.
